# The effects of synbiotic supplementation on serum inflammatory markers and edema volume in breast cancer survivors with lymphedema

**DOI:** 10.17179/excli2019-1876

**Published:** 2020-01-02

**Authors:** Saeideh Vafa, Shahpar Haghighat, Leila Janani, Ali Saneei Totmaj, Mehraban Navaei, Ali Amirinejad, Hadi Emamat, Zahra Salehi, Mitra Zarrati

**Affiliations:** 1 Department of Nutrition, School of Public Health, Iran University of Medical Sciences, Tehran, Iran; 2 Breast Cancer Research Center, Motamed Cancer Institute, ACECR, Tehran, Iran; 3 Department of Biostatistics, School of Public Health, Iran University of Medical Sciences, Tehran, Iran; 4 Student Research Committee, Department of Clinical Nutrition and Dietetics, Faculty of Nutrition Sciences and Food Technology, National Nutrition and Food Technology Research Institute, Shahid Beheshti University of Medical Sciences, Tehran, Iran; 5 Immunology Department, School of Medicine, Tehran University of Medical Sciences, Tehran, Iran

**Keywords:** inflammation, breast cancer, lymphedema, probiotic, prebiotic, caloric restriction

## Abstract

Breast cancer-related lymphedema (BCRL) is one of the most common complications of breast cancer treatments, which may be exacerbated by obesity. Dysbiosis may negatively impact the management of obesity and lymphedema by increasing inflammation. The objective of this study was to assess the effects of supplementation with synbiotics on inflammatory markers, serum leptin concentration and edema volume in overweight and obese BCRL women following a low-calorie diet (LCD). In a randomized double-blind controlled clinical trial, 88 breast cancer survivors with lymphedema were supplemented once a day for 10 weeks with either a synbiotic or a placebo capsule. Both groups were under a low-calorie diet (LCD). At the end of the study, synbiotic supplementation resulted in a significant reduction in leptin (P=0.003) and TNF-α (P=0.039) between the study groups. Besides, edema volume was significantly reduced within the synbiotic group after the intervention. We did not observe any significant effects of the synbiotic supplementation in hs-CRP, and IL-1β between the study groups (P=0.550, P=0.118 respectively). Conclusively, synbiotic supplementation along with an LCD program in breast cancer survivors with lymphedema had beneficial effects on the concentration of serum inflammatory markers and edema volume.

## Introduction

Breast cancer (BC) is an important health concern in women worldwide (Coughlin and Ekwueme, 2009[11]). Due to advances in cancer treatment and early detection, the five-year relative survival rate for females diagnosed with BC has increased to 89.7 % (American Cancer Society, 2018[3]). However, many of these patients face chronic complications of BC treatments, such as lymphedema (Erickson et al., 2001[16]). Lymphedema is the most prevalent and distressing symptom for patients with breast cancer after surgery and/or radiation (Wanchai et al., 2016[55]). 

Breast cancer survivors may experience lymphedema complications for months or years after cancer treatment (Norman et al., 2009[33]). Breast cancer-related lymphedema (BCRL) incidence has been reported to be 21.4 % (DiSipio et al., 2013[14]) and is characterized by swelling, heaviness, pain, limb use limitation, and decreased quality of life (Hormes et al., 2010[20]; Dayan et al., 2018[12]). The lymphatic system is critically important in maintaining tissue fluid homeostasis and regulating the inflammatory response by improving drainage of extravasated fluid, thus reducing edema formation and levels of pro-inflammatory mediators include tumor necrosis factor-α (TNF-α) (Schwager and Detmar, 2019[42]). 

Lymphatic injury due to cancer treatments leads to the disruption of lymph flow. Lymphatic fluid stasis may cause increased inflammatory mediators such as TNF-α, fat deposition and progressive fibrosis (Gousopoulos et al., 2016[17], Tashiro et al., 2017[49]). Beside, TNF-α plays the main role in inflammation-induced lymphatic contractile dysfunction (Chen et al., 2017[10]). Studies have implicated that chronic inflammation is a hallmark in the pathophysiology of lymphedema (Ly et al., 2017[25]; Rockson et al., 2018[37]).

Many studies indicated that obesity and also overweight may predispose women to lymphedema and may affect the response to treatment (DiSipio et al., 2013[14]; Ly et al., 2017[25]; Helyer et al., 2010[18]; Hespe et al., 2017[19]; Rockson et al., 2018[37]). Moreover, obesity has a positive correlation with lymphedema severity (Ridner et al., 2011[35]; Helyer et al., 2010[18]). In contrast, weight loss programs have effectively improved the treatment of BCRL (Shaw et al., 2007[44][45]). An increase in serum leptin concentration has been reported in obese patients (Minocci et al., 2000[29]; Van Dielen et al., 2001[51]). Leptin enhances the expression of pro-inflammatory cytokines, such as TNF-α (Bulló et al., 2003[9]). Since obesity is a major risk factor for lymphedema (Vignes et al., 2007[54]; Mehrara and Greene, 2014[28]), the increased level of leptin in overweight and obese patients, can exacerbate inflammation and increase the edema severity (Ly et al., 2017[25]). Anti-inflammatory and anti-fibrotic agents seem to be two key factors in preventing the progression of this disorder (Schaverien and Aldrich, 2018[41]; Hespe et al., 2017[19]; Rockson et al., 2018[37]). 

Dysbiosis or gut microflora alteration is not uncommon among overweight and obese individuals (Brown et al., 2012[8]), which may cause systemic inflammation (Le Chatelier et al., 2013[24]; Verdam et al., 2013[53]). Probiotics are living and non-pathogenic bacteria with helpful effects for improving or maintaining the microbiome which can modify the microflora (Alard et al., 2016[1]; Wischmeyer et al., 2016[56]). Synbiotics refer to food ingredients or dietary supplements combining probiotics and prebiotics, non-digestible fiber compounds that stimulate the growth of beneficial bacteria, in the form of synergism (Pandey et al., 2015[34]). Further, probiotics may contribute to improved homeostasis and decreased side effects related to anti-cancer therapies (Mego et al., 2013[27]). Moreover, it has been shown that decreased inflammation may occur by reducing the production of pro-inflammatory cytokines, including interleukin‐1 (IL-1) and TNF-α (McLoughlin et al., 2017[26]). On the other hand, the intestinal microbiome dysbiosis has been shown to affect the development of lymphedema through complicated biological mechanisms including downregulation of pro-inflammatory cytokines such as TNF-α (Amdekar et al., 2012[2]; Archer et al., 2015[4]; Solanki et al., 2015[46]).

We are aware of no study that has examined the effect of synbiotic supplementation on inflammatory profiles and edema volume in BCRL. The current study was therefore performed to investigate the effects of synbiotics on the inflammation status and edema volume of breast cancer survivors with lymphedema following a low-calorie diet. The result of this research may provide new strategies for lymphedema management.

## Materials and Methods

### Study design 

Women with BC related lymphedema who were treated by complex decongestive therapy (CDT) were recruited in this randomized double-blind controlled clinical trial. Lymphedema therapy was carried out in two phases, including an intensive phase that is performed in the clinic and a maintenance phase which is achieved by the patient and family at home to stabilize edema reduction. This study was conducted in the maintenance phase to assess the effect of the interventions on changing limb volume.

Our study was approved by the Ethics Committee of Iran University of Medical Sciences and followed the Declaration of Helsinki and Good Clinical Practice guidelines. All participants signed a written informed consent before study enrollment. This research was registered at the Iranian Registry of Clinical Trials (IRCT2017092023861N7).

### Participants 

Eighty-eight women with unilateral arm lymphedema diagnosed by a lymph therapist were recruited to the study at Seyed Khandan Rehabilitation Clinic at Tehran, Iran from October 2017 to November 2018. Inclusion criteria were age between 18 and 65 years, BMI 25 - 40 kg/m^2^, stage 1 or 2 of lymphedema, completed surgery and adjuvant treatment completion (except hormone therapy/aromatase inhibitors) at least six months before enrollment. Patients with a history of probiotic supplements or anti-inflammatory drugs consumption and participating in weight loss programs in the last 6 months, were excluded from the study. Other exclusion criteria were included BC recurrence or metastasis, infection, smoking and alcohol consumption, autoimmune diseases, serious food allergies, mental illness, endocrine diseases including thyroid disorders, diabetes, gastrointestinal problems, taking multivitamin-mineral and omega-3 up to one month before the start of the study, continuous and heavy physical activity and history of diseases leading to edema such as heart, kidney, or liver failure.

### Randomization and allocation

Participants were randomly assigned to the synbiotic supplementation group (n=44( or the placebo group (n=44). All participants received a low-calorie diet from the beginning to the end of the intervention.

For randomization, permuted-block randomization with a block size of 4 was performed by the statistician consultant using the online site (www.sealedenvelope.com). To apply the concealment in the randomization process, unique codes were assigned to the supplement boxes, which were generated by the software. Randomization and allocation were concealed from investigators, participants, and analyzers until the statistical analysis was completed.

### Weight-reduction program

In this study, the individual dietary program was based on baseline weight and the subject's food records to produce an energy deficit of 500 to 1000 kcal/day from estimated energy requirements throughout the study. No participant was recommended a daily intake <1200 kcal. The participant's energy requirement was calculated according to the Mifflin-St. Jeor equations. The recommended composition of the diet was 55 % to 60 % of the energy from carbohydrates, 10 % to 15 % from protein and 20 % to 35 % from total fat.

### Intervention

The synbiotic supplements (Lacto Care, Zist Takhmir Co., Tehran, Iran) contained 10^9 ^colony-forming units (CFU) per gr (CFU/g) beneficial bacteria such as *Lactobacillus casei, Lactobacillus acidophilus, Lactobacillus rhamnosus, Lactobacillus bulgaricus, Bifidobacterium breve, Bifidobacterium longum, Streptococcus thermophiles*, and 38.5 mg fructo-oligosaccharides. The placebo was a capsule similar in appearance, weight, smell, and packaging, containing lactose (Zist Takhmir Co., Tehran, Iran).

The women in the synbiotic or placebo groups took a daily capsule for 10 weeks. They were asked to keep the study capsules refrigerated (between 2 and 7˚ C) throughout the study. They were also requested not to take any probiotic-containing food, probiotic yogurt or its products in 14 days before (2 week run-in period) and throughout the study. Compliance with consumption of capsules was monitored once a week through phone calls and confirmed by counting the capsules in face to face interviews after 5 weeks of intervention.

### Assessment of variables

Demographic and clinical characteristics were collected by personal interview and the medical report. They were recorded in a checklist. Anthropometric indices were measured for all participants at baseline and after 10 weeks of intervention. Height and weight were measured without shoes and in light clothing using a digital scale (Seca, Hamburg, Germany). Body mass index (BMI) was calculated using formula weight in kg divided by height in m^2^. Waist circumference was measured at the narrowest part of the torso. 

Lymphedema volume was measured using the water displacement method (submerging the healthy limb and then the affected limb in a water tank up to 2 cm below the armpit). Edema volume was calculated as the volume difference between affected and unaffected arms in milliliters. All measurements conducted by an experienced examiner who was unaware of the study-group assignments.

Nutrient intake of the participants at the beginning and end of the study, based on a 3-day food record (two weekdays and one weekend day), were calculated by Nutritionist IV software (First Databank, San Bruno, Calif, USA) modified for Iranian foods. Physical activity was assessed using an International Physical Activity Questionnaire (IPAQ) by interview (Vasheghani-Farahani et al., 2011[52]). 

Blood samples were taken after 8-12 hour fasting at baseline and the end of the 10 weeks intervention. Serum samples were separated by centrifugation (Hettich D-78532, Tuttlingen, Germany) at 3500 rpm for 10 min and stored at -80 °C until assayed. Serum concentrations of leptin, IL-1β, and TNF-α were measured using ELISA kits (eBioscience, US) and hs-CRP was measured by an immunoturbidimetric assay (Pars Azmoon kit, Tehran, Iran).

### Sample size

The primary outcome of this study was TNF-α, since its effect had not been reported in similar studies. Based on the amount of Cohen's standard effect, assuming probability of a type I error of 5 % (α = 0.05) and a type II error of 20 % (β = 0.2; power = 80 %) and the Cohen standardized effect value of 0.65 (one effect at moderate level), also considering up to 10 % losses to follow-up, to detect the desired effect, the sample size was calculated 44 patients for each group.

### Statistical analysis

For continuous variables, the Shapiro-Wilk's test and a histogram were applied to ensure normality. All participants who were randomly assigned and completed an initial assessment were included in the final results by using an intention-to-treat analysis. For quantitative variables, the mean ± standard deviation or the median (first quartile - third quartile) and for qualitative variables, frequency (percent) was used.

To compare quantitative outcomes between the study groups, an independent samples t-test (for age, age at cancer diagnosis, dietary intakes, weight, BMI, fat percent, waist circumference and physical activity) or a non-parametric Mann-Whitney test (for time since cancer treatment, duration of lymphedema, tumor size, CDT course numbers, lymph nodes dissected, lymph nodes involved, edema volume, hs-CRP, IL-1β, TNF-α, and leptin) was used. Changes of outcomes within each group after the intervention compared to baseline values were assessed by a paired sample t-test or a non-parametric Wilcoxon rank signed test. Chi-square test or Fisher's exact test was used to compare qualitative factors (including lymphedema stage, surgery type, chemotherapy, radiotherapy, etc.) between the study groups. Delta (Δ) was used to show the differences in the variables before and after the intervention. We used ANCOVA to compare post-intervention outcomes between the study groups by adjustment on baseline values of each outcome and baseline BMI as the covariate. Spearman's rank correlation coefficient analysis was performed to evaluate the relationship between changes in body weight/waist circumference and the blood factors/edema volume difference. The level of significance for all statistical analyses was p < 0.05. All statistical analyses were performed using SPSS version 24 (IBM SPSS, Armonk, NY: 2016). Non-parametric ANCOVA analysis was performed with R software (package 'sm' in R-3.5.1 for windows).

Our approach for analyses can be considered as an intention-to-treat (ITT) analysis, since it includes every subject who was randomized according to randomized treatment assignment. It ignores noncompliance, protocol deviations, withdrawal and anything that happens after randomization.

## Results

### Participants' characteristics

**Figure 1:**
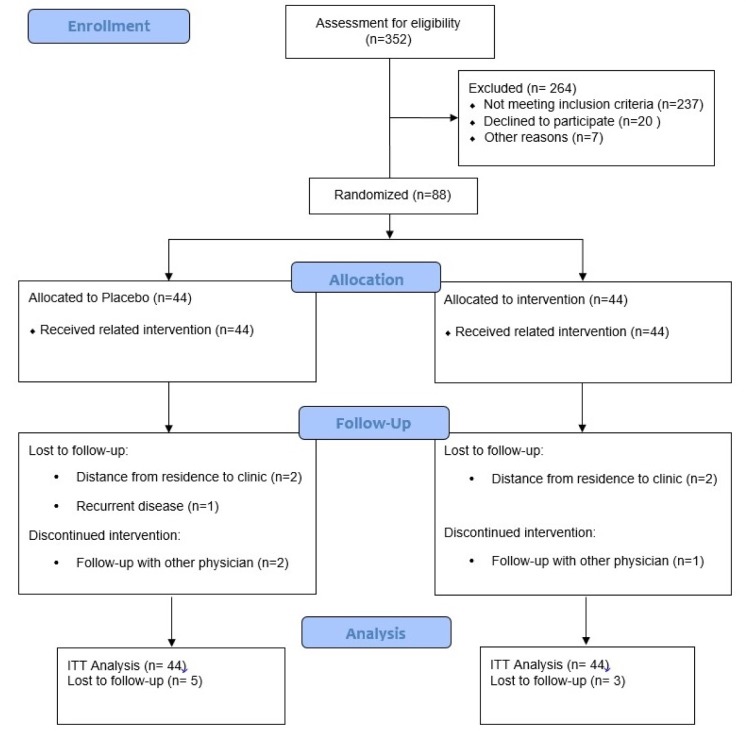
Consort diagram

As demonstrated in the study flow diagram (Figure 1(Fig. 1)), we recruited 352 participants. However, 264 subjects were excluded from the study because of not meeting the inclusion criteria. Of the 88 women enrolled in the study, 44 participants were randomized to the synbiotic group, and 44 were randomized to the placebo group. Eight participants (10 %) withdrew from the study following randomization: four patients because of distance from residence to the clinic, one patient because of recurrent disease and three patients because of follow-up with other physicians. Finally, 41 and 39 participants in the synbiotic and placebo groups completed the trial, respectively.

No side effects were reported following the supplementation of synbiotic in breast cancer survivors with lymphedema throughout the study.

Baseline demographic and clinical characteristics of lymphedema patients have been presented in Table 1(Tab. 1). The baseline mean (± SD) age of participants was 53.80 (± 9.16) years in the synbiotic group and 52 (± 7.95) in the placebo group. There was no significant difference between the two groups regarding demographic and clinical characteristics.

### Dietary intake

According to the 3-day food records obtained within each group before and after the intervention, there was no statistically significant difference in terms of energy, dietary macro- and micro-nutrient intakes between the study groups in baseline and after 10 weeks of intervention (Table 2(Tab. 2)). 

However, both synbiotic and placebo groups showed a significant within-group reduction in daily energy intake (P < 0.001, P < 0.001), carbohydrate (P < 0.001, P < 0.001), protein (P < 0.001, P =0.001), total fat (P = 0.001, P = 0.005), SFA (P = 0.001, P < 0.001), PUFA (P < 0.005, P < 0.001), MUFA (P = 0.003, P < 0.001), cholesterol (P=0.047, P=0.037), sodium (P=0.035, P=0.002) and beta-carotene (P=0.049, P=0.001) respectively (Table 2(Tab. 2)).

### Anthropometry and physical activity 

The mean values of weight (kg), BMI (kg/m^2^), body fat percent (%), waist circumference (cm) and physical activity (min/week) at baseline and after 10 weeks in synbiotic and control groups are depicted in Table 3(Tab. 3). The statistic tests showed no significant difference between the study groups after the intervention. Within groups changes showed significant statistical differences (P< 0.005) in synbiotic and control groups regarding mean value of weight (-2.48 ± 3.03 kg, -1.73 ± 2.26 kg), BMI (-0.95 ± 1.14 kg/m^2^, -0.68 ± 0.90 kg/m^2)^, body fat percent (-1.14 % ± 1.37, -0.83 % ± 1.09) and waist circumference changes (-3.89 ± 3.26 cm, -3.84 ± 3.60 cm), respectively. 

The physical activity level significantly increased within each group [17.0 (3.25, 30.50) min/week and 21.0 (7.0, 33.0) min/week in synbiotic and control groups respectively, P< 0.001]. The intention-to-treat (ITT) analysis did not show different results between the study groups.

### Edema volume measurement

As shown in Table 4(Tab. 4), there was a significant reduction in edema volume in the synbiotic group after the 10 week intervention (-37.26 percent, P< 0.001). However, according to the ANCOVA tests adjusted for baseline edema volume and pre-intervention BMI, there were no significant differences between the study groups after the intervention (P=0.180).

### Inflammatory markers 

Baseline measures of inflammatory markers were not significantly different between the study groups, except hs-CRP [median (IQR): 3.20 (3.75), 1.85 (1.88) in synbiotic and placebo group respectively, P= 0.022]. In the synbiotic group after 10 weeks of intervention, hs-CRP (-3.12 percent, P=0.032), IL-1β (-8.37 percent, P=0.018), and leptin (-25.58 percent, P=0.026) decreased significantly (Table 4(Tab. 4)). Initial analysis showed no significant change in serum TNF-α levels following supplementation with synbiotic (Table 5(Tab. 5)) but ITT analysis revealed lower TNF-α values in the synbiotic group following intervention (P=0.039). Data in Table 5(Tab. 5) demonstrates that 10 weeks of synbiotic supplementation significantly reduced TNF-α and leptin between the study groups (Adjusted P= 0.039; P= 0.003, respectively).

The correlation of changes in serum hs-CRP with body weight (r=0.420, P=0.006) and waist circumference (r=0.376, P=0.018) alterations in the synbiotic group was statistically significant. Also, edema volume changes significantly correlated with body weight (r=0.326, P=0.038) and waist circumference (r= 0.330, P=0.035) in the synbiotic group (Table 6(Tab. 6)).

## Discussion

This is the first interventional trial that investigates the possible beneficial role of synbiotic supplementation on inflammatory markers (TNF-α, IL-1β, hs-CRP), serum leptin concentration, anthropometric parameters, and edema volume in BCRL patients. 

Our results revealed that synbiotic supplementation for 10 weeks reduced edema volume, the serum concentration of hs-CRP, IL-1β and leptin in overweight and obese patients with BCRL. Furthermore, the synbiotics group had significantly lower levels of serum leptin and TNF-α concentration compared to placebo after the intervention.

A significant improvement was seen in anthropometric indices including body weight, BMI, fat percent and waist circumference within groups at the end of the intervention which is the predictable consequence of the low-calorie diet prescribed to all patients. The anthropometric changes were not significant between the study groups because the simultaneous occurrence of cancer, lymphedema and obesity increased the patient's inflammation (Kolb et al., 2016[23], Zahid et al., 2016[58]) to the level that we could not observe any significant effects for synbiotics in these cases. Also, in a meta-analysis study was shown that probiotic supplementation (3 to 12 weeks) has a small size effect on the reduction of anthropometric factors (Borgeraas et al., 2018[7]).

Reduction in excess edema volume after synbiotic supplementation is a new finding that has not been reported elsewhere. The percentage of edema reduction was 37.36 % and 20.75 % in the synbiotic and placebo groups, respectively. The lack of significant differences between the study groups after the intervention could indicate the positive effects of the weight-loss diet on this marker. In the study by Shaw et al. 12 weeks prescription of a weight-loss diet, led to a significant reduction in weight, BMI, and edema volume in BCRL patients (Shaw et al., 2007[44]) Probiotics with their weight-lowering characteristics can promote this positive effect. Obesity is one of the well-known factors in the onset and progression of lymphedema and obese individuals have a decreased lymphatic transport capacity (Yoon et al., 2018[57]; Savetsky et al., 2014[39]). 

In different studies, the prognostic effect of high BMI in lymphedema incidence (Yoon et al., 2018[57]; Iyigun et al., 2018[21]; Helyer et al., 2010[18]) and the predictive effect of weight loss accompanied by complete decongestive therapy in edema reduction (Shaw et al., 2007[44][45]; Duyur Cakit et al., 2019[15]) have been confirmed. 

Alleviated hs-CRP serum levels in the synbiotic group was another finding of this study. This beneficial effect was also seen in another study performed on diabetic hemodialysis patients. After 12 weeks of intervention, serum hs-CRP was significantly lower in the synbiotics group (Soleimani et al., 2017[47]). Obesity by activation of the c-Jun N-terminal kinase, nuclear factor-kappa B, and protein kinase R pathways induce the inflammatory response (Solinas et al., 2010[48]; Nakamura et al., 2010[31]). Lack of significant differences in hs-CRP serum levels between the study groups after the intervention may be because of the synergistic effects of cancer/chemotherapy and obesity on inflammation in our study (Kolb et al., 2016[23]; Zahid et al., 2016[58]).

The results of the current study showed a significant reduction in serum IL-1β in both groups after the intervention (-8.37 %; control = -5.01 %), but the non-significant differences of this variable between groups are probably due to the beneficial effects of the weight loss program on this cytokine, owing to the fact that a decrease in body fat during weight loss reduces the production of IL-1β (Kirchner et al., 2014[22]). Shadnoush et al. reported that 8 weeks of consumption of *bifidobacterium *and *lactobacillus *in the form of probiotic yogurt, decreased serum IL-1β concentration in inflammatory bowel disease patients compared to control (Shadnoush et al., 2013[43]). No weight loss program was performed in their study that could explain why the differences in serum IL-1β were significant after probiotic intake. 

Obesity leads to impairment and imbalance in gut microbiota called dysbiosis which weakens intestinal integrity accompanied by lipopolysaccharide (LPS)-related endotoxemia. LPS is a large molecule that exists in the cell wall of gram-negative bacteria. LPS stimulates the activation of the transcription nuclear factor-kappa B (NF-κB) by binding to toll-like receptor 4 (TLR4) in intestinal epithelial cells. NF-κB upregulates gene expression of pro-inflammatory cytokines such as TNF-α, IL-1β, and IL-6 which mediate a rise in hs-CRP, inflammation, and consequently edema (Neyrinck et al., 2016[32]; Torres et al., 2018[50]; Dinarello, 2009[13]). Also, producing short-chain fatty acids by probiotics prevents the synthesis of hepatic CRP that results in a reduction in its serum levels (Badehnoosh et al., 2018[5]). 

We demonstrated that synbiotic supplementation for 10 weeks in BCRL patients led to a significant reduction in serum leptin levels compared to baseline and control group. This effect was also seen in studies on overweight and obese individuals (Sanchez et al., 2014[38]; Zarrati et al., 2014[59]), but it was not in line with the study by Mobini et al. Supplementation with *Lactobacillus reuteri* in diabetic patients for 12 weeks, could not change leptin (Mobini et al., 2017[30]). Our supplements contain diverse strains of probiotics compared to that study. Each strain of probiotics may exert a special function (Pandey et al., 2015[34]), which may be responsible for the inconsistency of the results. Several mechanisms can explain the beneficial effects of probiotics serum leptin levels. Probiotics have a critical role in leptin secretion regulation (Behrouz et al., 2017[6]). Leptin increases the production of TNF-α in monocytes (Scarpellini and Tack, 2012[40]; Zhou et al., 2011[60]). Any factor that reduces the leptin level can also reduce the levels of TNF-α. Therefore, in our study, a decrease in the leptin serum level consequently reduced the TNF-α serum level in the synbiotic group compared with the placebo. Despite a significant decrease in TNF-α serum level, because of multiple outcomes and the possibility of significant correlation due to multiple testing, this factor should be interpreted with caution. An adjusted significant level can be confirmed in the next and larger studies.

In spite of the potential strengths of our study, such as using a probiotic intervention for the first time among BCRL patients, this study has some limitations. Although the synbiotics supplementation may affect fecal bacterial load, we could not assess this correlation because of budget limitations. Also, we did not consider a control group receiving placebo capsules without any low-calorie diet. So, we can not conclude if the weight reduction program had a synergistic effect along with the synbiotic capsules or not. Evaluation of the gene expression of inflammatory cytokines in future researches may provide valuable evidence in this field. 

## Conclusion

In conclusion, 10 weeks of synbiotic supplementation along with an LCD program among BCRL patients resulted in a significant reduction in serum levels of TNF-α and leptin compared with the control group. Also, edema volume was significantly reduced within the synbiotic group after intervention. However, no significant differences were found in either of the anthropometric variables, IL-1β, and hs-CRP between the study groups. Weight reduction and synbiotic product consumption in the daily diet of lymphedema patients may be suggested as effective and safe interventions in lymphedema management.

## Notes

Shahpar Haghighat and Mitra Zarrati (Faculty of Nutrition, School of Public, Iran University of Medical Sciences, Tehran, Iran; Tel: +982186704814, Fax: +982186704814, E-mail: Zarrati_ms@yahoo.com, zarrati.m@iums.ac.ir) contributed equally as corresponding authors.

## Acknowledgement

We would like to gratefully thank the participants for their support in the study. We are also thankful to Namdar laboratory and Zist Takhmir Company and all patients that participated in this study.

## Funding

This study was supported by Iran University of Medical Sciences (IUMS) with a 96-02-27-31433 grant number. 

## Conflict of interest

The authors declare that they have no conflict of interest.

## Figures and Tables

**Table 1:**
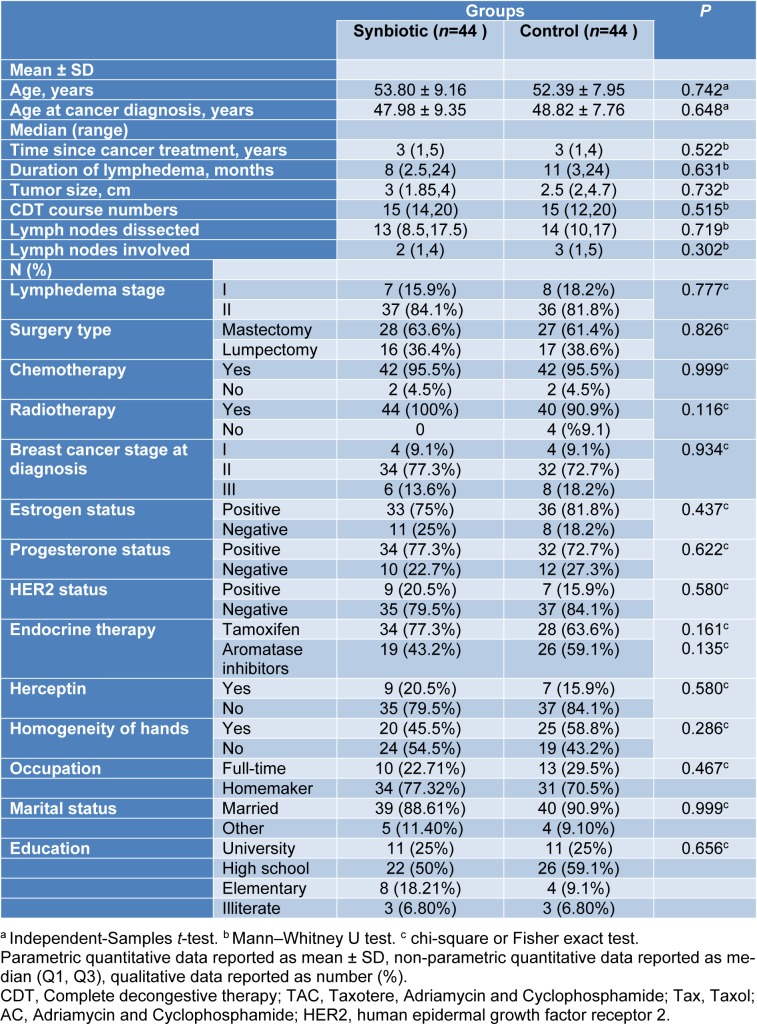
Clinical and demographic characteristics of participants at baseline

**Table 2:**
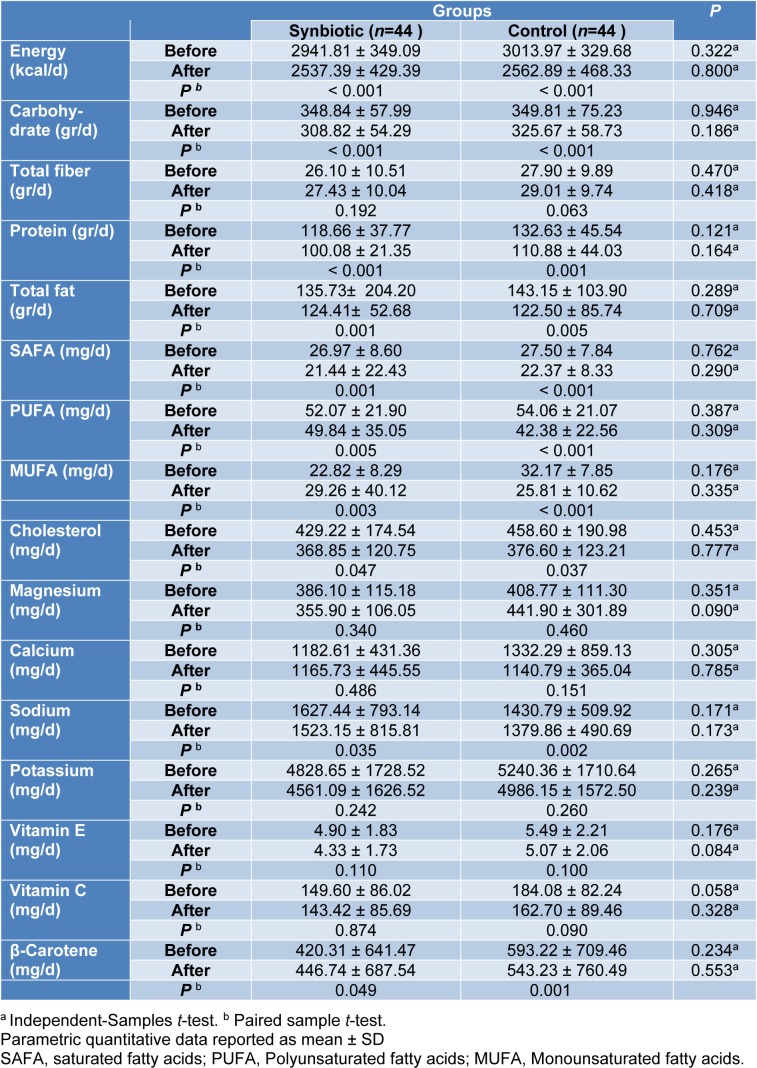
Dietary intakes of participants throughout the study

**Table 3:**
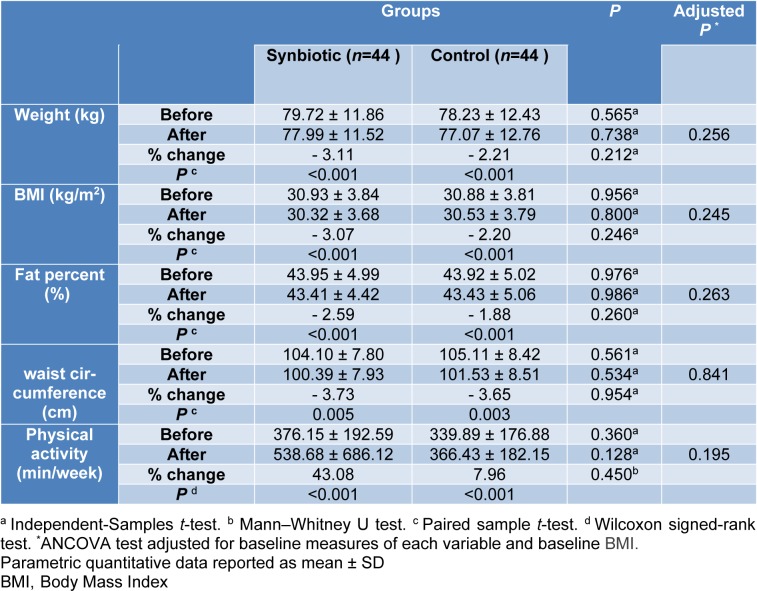
Anthropometrics and physical activity characteristics in two groups before and after intervention

**Table 4:**
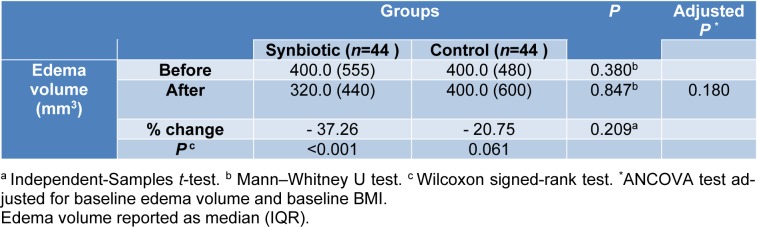
Participants edema volume characteristics in two groups before and after intervention

**Table 5:**
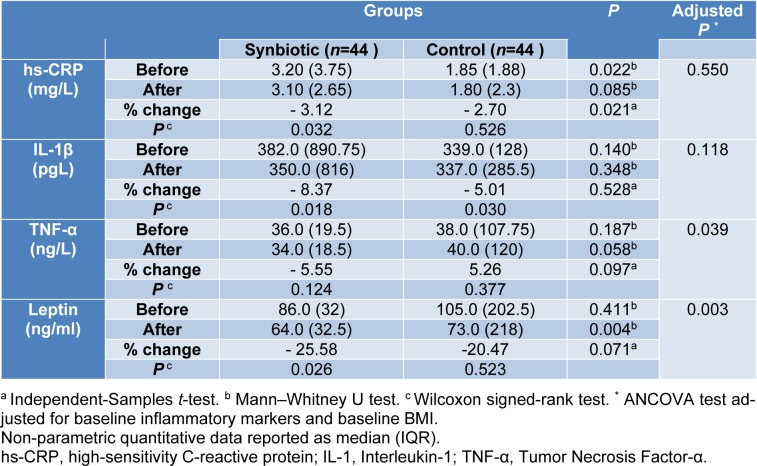
Inflammatory markers in two groups before and after intervention

**Table 6:**
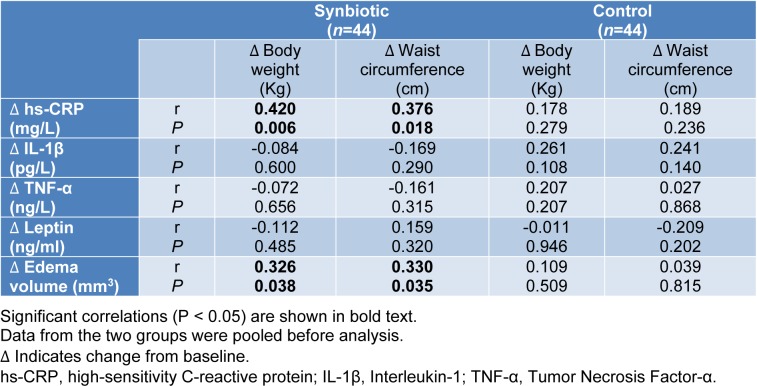
The correlation of inflammatory marker and edema volume with body weight and waist circumference in synbiotic and control groups
